# Building a brain under nutritional restriction: insights on sparing and plasticity from *Drosophila* studies

**DOI:** 10.3389/fphys.2014.00117

**Published:** 2014-03-26

**Authors:** Elodie Lanet, Cédric Maurange

**Affiliations:** Aix Marseille Université, CNRS, IBDM UMR 7288Marseille, France

**Keywords:** Drosophila, neuroblasts, nutritional physiological phenomena, brain development, brain sparing, brain plasticity, neural stem cell (NSC), temporal patterning

## Abstract

While the growth of the developing brain is known to be well-protected compared to other organs in the face of nutrient restriction (NR), careful analysis has revealed a range of structural alterations and long-term neurological defects. Yet, despite intensive studies, little is known about the basic principles that govern brain development under nutrient deprivation. For over 20 years, *Drosophila* has proved to be a useful model for investigating how a functional nervous system develops from a restricted number of neural stem cells (NSCs). Recently, a few studies have started to uncover molecular mechanisms as well as region-specific adaptive strategies that preserve brain functionality and neuronal repertoire under NR, while modulating neuron numbers. Here, we review the developmental constraints that condition the response of the developing brain to NR. We then analyze the recent *Drosophila* work to highlight key principles that drive sparing and plasticity in different regions of the central nervous system (CNS). As simple animal models start to build a more integrated picture, understanding how the developing brain copes with NR could help in defining strategies to limit damage and improve brain recovery after birth.

## Introduction

One of the most disrupting conditions a multicellular organism can encounter during development is nutrient restriction (NR). In response, many tissues undergo a decline in both cell proliferation and growth rate leading to undersized adults. For example, *Drosophila* flies may only reach half of their normal size when grown on a deprived diet, and tissues such as the eye or wings undergo a consistent 25% reduction in cell numbers (Brogiolo et al., 2001; Puig et al., 2003; Hietakangas and Cohen, 2009; Lanet et al., 2013). In mammals, reduced food availability to the mother or placental insufficiency leads to smaller new-borns. However, it is often observed that such newborns possess a proportionally larger head than then rest of the body, a phenomenon known in human as asymmetric intra-uterine growth restriction (IUGR) (Cox and Marton, 2009). Thus, when confronted with nutrient limitation, especially during late fetal stages, the brain does not reduce its size isometrically with the rest of the body. This brain-sparing phenomenon reflects a survival strategy that preferentially protects more critical organs, at the expense of others, ensuring that both cell size and numbers attain near-normal proportions independently of nutritional conditions. However, beyond global sparing, numerous studies in various mammalian models depict a complex picture with NR-induced structural alterations, the nature and strength of which are region-specific (Morgane et al., 2002; Alamy and Bengelloun, 2012). Moreover, while some of these alterations recover on return to a normal diet after birth, growing evidence suggests that early nutritional stress leaves permanent traces responsible for abnormal cognitive, behavioral, and psychiatric outcomes later in life (Hulshoff Pol et al., 2000; Rehn et al., 2004; Roza et al., 2008; De Rooij et al., 2010).

Despite intensive studies, there is no integrated understanding of the laws that determine how the different regions of the developing brain respond to nutritional stress that might help to predict the most adverse outcomes. Here, we review recent studies performed in the fruitfly *Drosophila* that identify principles governing development of the central nervous system (CNS) under NR.

## *Drosophila*: a model to investigate CNS development when nutrients are scarce

**Figure 1:**
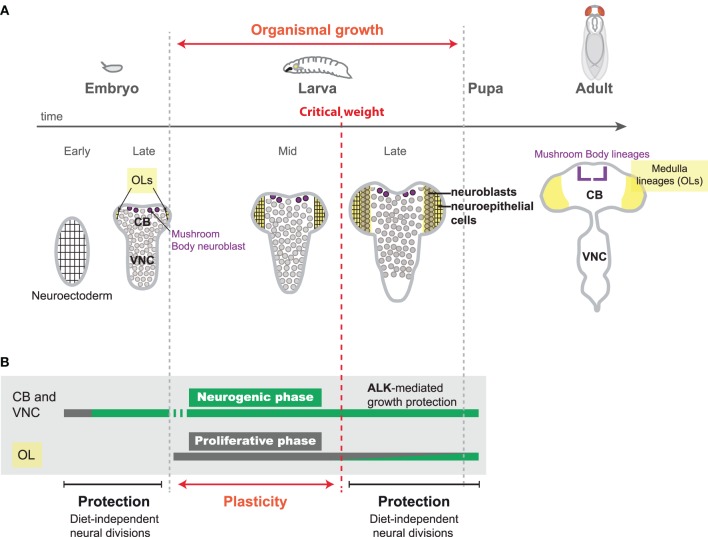
**Neural proliferation during *Drosophila* development. (A)** The main period of growth in insect development occurs during larval stages. Upon feeding, larvae continuously and rapidly grow, reaching a so-called “critical weight.” From this moment, pulses of the steroid hormone ecdysone are produced, triggering pupariation 24 hr later. Neural development starts during embryogenesis and is completed before adult hatching. During early embryonic stages, the neuroectoderm amplifies by symmetric divisions (proliferative phase). Neuroblasts of the central brain (CB) and the ventral nerve cord (VNC) delaminate from the neuroectoderm and undergo a series of asymmetric divisions to produce neurons (neurogenic phase). During larval stages, CB and VNC neuroblasts continue to produce a large number of neurons. In the optic lobes (OLs) (yellow) a neuroepithelium (rectangular cells) starts expanding (proliferative phase) as the larvae starts feeding. When the larva reaches the “critical weight,” pulses of ecdysone are produced that promote the conversion of the neuroepithelium in neuroblasts (circular cells), which generate neurons (neurogenic phase). **(B)** During embryonic stages, neural divisions are not influenced by dietary conditions, as embryos are isolated from the environment and possess their own nutritional reserves. Sparing of neural growth and division also occurs after reaching critical weight when the animal possesses large amounts of endogenous nutritional reserves to fuel biogenesis. In the CB and VNC, neuroblast growth is additionally supported by insulin-independent growth Alk activity (Cheng et al., 2011). In contrast, the first stages of larval development provide a window of plasticity to adapt the neuronal content to nutritional conditions.

Over the last 25 years, CNS development has been extensively studied in *Drosophila*, and this model organism has had a lead role in our understanding of the molecular and cellular principles governing the building of a functional brain. The *Drosophila* CNS is much simpler than its mammalian counterpart. For the purpose of this review, we will subdivide it in three main regions: (i) the ventral nerve cord (VNC) or insect equivalent of the spinal cord, (ii) the central brain (CB), including a set of lineages that form the mushroom bodies, and (iii) the visual system also called the optic lobes (OLs) (Figure 1A). The three regions develop from a small pool of self-renewing, asymmetrically dividing neural stem cells (NSCs), called neuroblasts. Neuroblasts from the VNC and CB possess a neuroectodermal origin. During embryogenesis, an invariant and well-characterized number of neuroblasts delaminates from the neuroectoderm to undergo a series of asymmetric divisions, budding-off different types of neurons and glia. Together, they form a rudimentary CB and VNC necessary for larval life (Figure 1A). After a period of quiescence at the end of embryogenesis, most of these neuroblasts reenter the cell cycle during early larval stages and generate the larger part of their lineage post-embryonically. In contrast to VNC and CB neuroblasts, neuroblasts from the medulla region of the OLs are generated post-embryonically from a neuroepithelium in a way that resembles the neuroepithelial-to-radial glia conversion in the mammalian cortex (Farkas and Huttner, 2008; Brand and Livesey, 2011). The medulla neuroepithelium first proliferates and expands during the first two thirds of larval stages. During the last larval stage (L3), progression of a proneural wave converts all neuroepithelial cells into asymmetrically dividing neuroblasts, each of them generating a lineage that will be assembled in a medulla unit (Egger et al., 2007; Yasugi et al., 2008; Lanet et al., 2013) (Figure 1A). In all regions of the CNS, neurogenesis terminates during metamorphosis when neuroblasts either undergo cell-cycle exit or apoptosis (Maurange et al., 2008; Siegrist et al., 2010).

The embryo is a closed system and possesses its own nutritional reserves. It is therefore protected against post-fertilization NR which do not perturb the number of VNC and CB neuroblasts and the size of their embryonic lineage (Doe, 1992) (Figure 1B). However, larval stages are highly dependent on nutritional conditions (Figure 1B). Food abundance during larval stages determines the size of most adult organs and can easily be altered for experiments (Mirth and Shingleton, 2012). Consistently, larvae grown on a suboptimal protein diet exhibit a delayed development and give rise to much smaller adults (Hietakangas and Cohen, 2009). Moreover, if feeding is completely abolished during the last third of larval development, the larva will stop growing but its development proceeds normally, leading to downsized adults. In the latter case, pulses of the steroid hormone ecdysone that are produced once the larva has attained a so-called “critical weight,” maintain developmental progression and commit the larva to metamorphosis a few hours later (Figure 1A) (Mirth and Shingleton, 2012). Although the larva stops growing whenever it is starved, a recent study has shown that starvation after reaching “critical weight” does not prevent the CNS from growing at an almost normal rate (Cheng et al., 2011). Therefore, protection mechanisms preferentially spare the growth of the different lineages of the VNC, CB, and OLs at least during the later part of larval development. Interestingly, this observation is reminiscent of the brain-sparing phenomenon observed during the last third of pregnancy in mammals.

## Why brain sparing? developmental constraints in the brain under construction

The brain-sparing effect observed in both mammals and flies suggests that the building of a functional CNS may not withstand a reduction of cell size and cell number in response to dietary restriction, as other organs may do. Two characteristics of the nervous system are the large size of its cells and their extreme diversity. Although reaching a large size is a critical requirement for normal function of both glia and neurons (Cotter et al., 2001; Lloyd, 2013), it seems equally essential for normal brain function to protect its cell composition. In this review, we will concentrate on the mechanisms that regulate NSC growth and mitotic activity in *Drosophila* ensuring that the normal repertoire of neurons and glia are produced.

**Figure 2:**
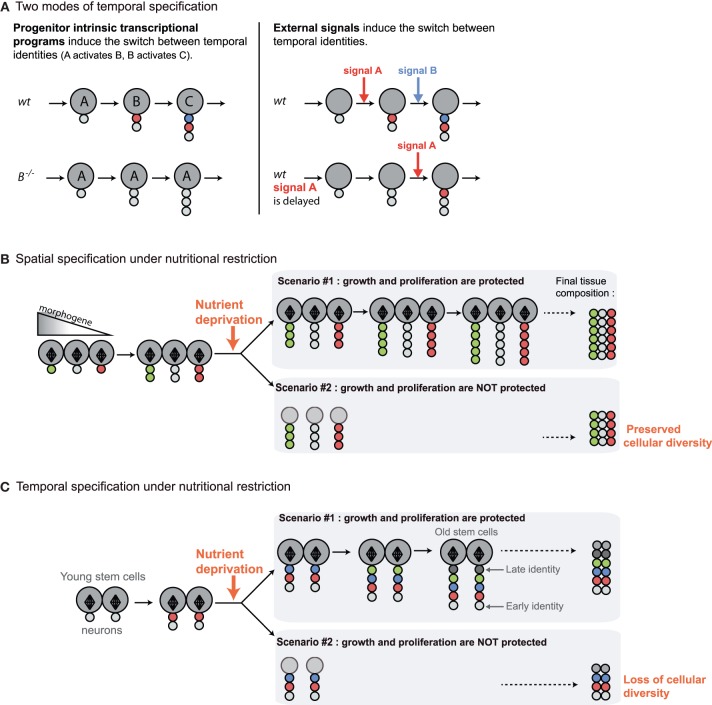
**Distinct mechanisms for promoting cell diversity during development imply distinct responses to nutrient deprivation**. In the subsequent figures, large circles represent neuroblasts and small circles, their differentiated progeny. Diamond shapes represent mitotic spindles of actively dividing progenitors. The various colors in differentiated progeny depict different neuronal identities in the lineage. **(A)** Two modes of temporal patterning have been described in *Drosophila*. In most lineages, progenitor intrinsic transcriptional programs regulate the sequential expression of “temporal” transcription factors (A, B, C) that specify distinct neuronal identities in the successive neuroblast progeny. Mutations in temporal transcription factors block the sequential expression of late factors and lead to neuroblasts that continuously generate progeny of the corresponding identity. On the other hand, in other lineages such as in the mushroom bodies, the transitions between neuronal identities are triggered by external signals. When signals are delayed, transitions to subsequent neuronal identities are also delayed. **(B)** In most organs, cell diversity mainly relies on the spatial specification of progenitors by gradients of morphogens. Initial spatial identity is transmitted to progeny generated throughout development. Precocious cell cycle arrest in the face of NR will not affect cellular diversity in the tissue. **(C)** In the nervous system, temporal patterning ensures that neural progenitors generate different types of neurons as they progress through successive rounds of asymmetric divisions. Precocious cell cycle arrest in the face of NR may perturb the production of progeny with late identities and, consequently, to reduce neuronal diversity in the lineage. This suggests that tissues subjected to temporal patterning are likely to be protected against NR.

Over the last 15 years, seminal work in *Drosophila* has revealed novel strategies deployed in the CNS to increase cell diversity. In most organs, cell diversity mainly relies on the spatial specification of progenitors by early morphogenetic gradients (Dessaud et al., 2008) (Figure 2B). This system also applies to neural progenitors, but many of them encounter an additional “temporal” patterning strategy that allows them to generate different types of neurons or glia as they progress through successive rounds of asymmetric cell divisions (Jacob et al., 2008). The combination of spatial and temporal patterning largely accounts for the multiplicity of cell types observed in this tissue. In *Drosophila*, two modes of temporal patterning have been uncovered (Figure 2A). In a large subset of lineages, it is encoded by neuroblast-intrinsic genetic programs that involve the sequential expression of transcription factors endowing the successive progeny with different fates according to their birth-order (Maurange, 2012; Kohwi and Doe, 2013; Li et al., 2013a). In other lineages, the identity of neurons produced is specified by environmental cues that change during the course of development. For instance, it has recently been demonstrated that neurons in the mushroom bodies, a region of the CB involved in olfactory learning and memory, acquire different identities according to the levels of ecdysone produced at the time of their birth (Kucherenko et al., 2012; Wu et al., 2012; Kucherenko and Shcherbata, 2013). Therefore, whether it is intrinsically or extrinsically controlled, temporal patterning allows each asymmetrically dividing neural progenitor to generate a lineage comprising a vast repertoire of progeny with distinct fates.

However, temporal patterning implies that neuroblasts have to undergo a pre-determined number of asymmetric divisions in order to generate their full repertoire of neuronal/glial fates (neuroblast intrinsic mechanism) or to sustain asymmetric divisions for relatively long developmental periods during which various external cues are produced. In all cases, it can be predicted that protection mechanisms are present to prevent precocious exhaustion of neuroblasts asymmetric divisions in response to insufficient nutrient supply, and ensure that all fates are produced in a given lineage (Figure 2C). Therefore, there are specific constraints imposed by temporal specification and the necessity to multiply cell diversity in the CNS. In the following paragraphs, we will describe recently uncovered examples of sparing strategies that aim at protecting neuroblast asymmetric divisions in the developing *Drosophila* CNS. Surprisingly, these studies have also revealed striking examples of plasticity, reporting regions of the brain that adapt their neuronal content to nutritional conditions.

## Mechanisms protecting neural stem cell lineages during development

Two recent studies have revealed distinct protection mechanisms that allow neuroblasts to sustain their growth and mitotic activity during food deprivation.

### Insulin-independent maintenance of neuroblast growth and proliferation by the alk tyrosine kinase

**Figure 3:**
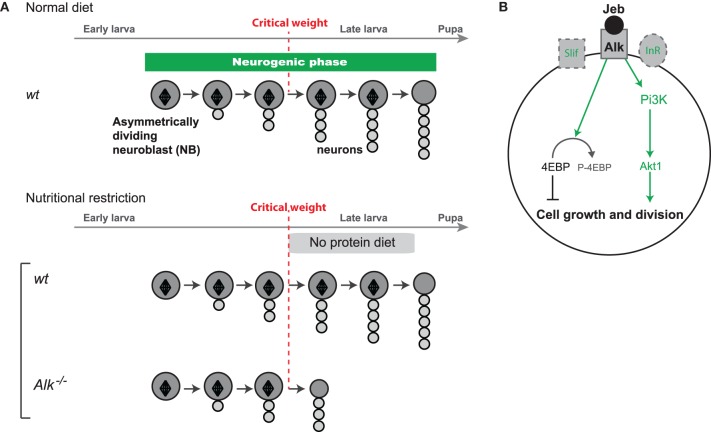
**Protection of neuroblast growth and proliferation by ALK**. **(A)** Alk tyrosine kinase protects growth and proliferation of neuroblasts in the absence of dietary nutrients after the critical weight. **(B)** Under nutrient restriction, the InR and amino-acid sensing pathways are not activated leading to growth arrest in most tissues. In neuroblasts, Alk is activated by its ligand Jelly-belly (Jeb) constantly produced by the niche. Subsequent constitutive activation of ALK can bypass the requirements for InR and the amino-acid sensing pathway leading to the activation of downstream effectors such as S6K and 4E-BP, that trigger biomass synthesis sustaining neuroblast mitotic activity and growth under NR.

During periods of starvation, systemic Insulin Growth Factors (IGFs), named Ilps in *Drosophila* (for Insulin-like peptides), decrease to low levels inducing global organismal growth arrest (Ikeya et al., 2002; Andersen et al., 2013). The discovery that the CNS of late larvae continues growing in such conditions suggested that brain-specific growth mechanisms could bypass the requirements of the Insulin Receptor (InR) pathway. Indeed, it was found that the constant activity in neuroblasts of Anaplastic lymphoma kinase (Alk), a tyrosine kinase like InR, could turn on downstream targets of the InR pathway in the absence of Ilps. This is made possible by the sustained production in the glial niche surrounding the neuroblast of Jelly-Belly, the Alk ligand, independently of dietary conditions (Figure 3A). Remarkably, Alk activation also suppresses the growth requirements for cellular amino-acid sensing by the Tor kinase and appears to regulate downstream effectors such as S6K and 4E-BP that control biomass synthesis (Figure 3B). Thus, in the CB and VNC of late larvae, activation of the biosynthetic pathways by ALK signaling ensures the continuous growth of neuroblast lineages regardless of nutritional conditions (Cheng et al., 2011) (Figure 3B).

Alk is a conserved protein known to be constitutively activated in some childhood neuroblastomas (Pugh et al., 2013), but its function during mammalian brain development has not been clearly investigated yet. A recent study in zebrafish indicates that Alk also plays a role during vertebrate neurogenesis. However, it is unknown whether the growth-sparing function is conserved (Yao et al., 2013). To date, stem or progenitor cells in other *Drosophila* tissues do not exhibit such growth protection, and instead critically require insulin and nutrient availability to be active (Shim et al., 2013).

Although neuroblast growth and activity is diet-insensitive during the last third of larval development thanks to ALK activity, this is not the case during early larval stages. Indeed, after embryonic development most neuroblasts (with the exception of mushroom body neuroblasts—see below) quit their quiescence state and resume dividing upon larval feeding, as the fat body senses the presence of amino-acids and sends signals that promotes Ilp production by the glial niche (Britton and Edgar, 1998; Chell and Brand, 2010; Sousa-Nunes et al., 2011). Thus, most neuroblasts in the VNC and CB switch during post-embryonic stages from a diet-sensitive to a diet-insensitive mode, but it is unclear exactly when and how Alk-mediated protection becomes operational.

### Coordination of the neurogenic phase with developmental stages possessing high nutritional reserves

We have recently shown that Alk-mediated growth boosting strategy may not operate in all parts of the CNS (Lanet et al., 2013). Indeed, in contrast to VNC and CB neuroblasts, those from the medulla of in the OLs decrease in size under food deprivation. However, medulla lineages are smaller than their VNC and CB counterparts (~20 cells vs. ~100 cells) and medulla NBs retain the ability to generate their entire lineage in the absence of dietary nutrients.

The diversity of neurons generated by each medulla neuroblast depends on an intrinsic temporal patterning system encoded by a well-described series of transcription factors, albeit distinct from the one described in embryonic neuroblasts of the VNC (Li et al., 2013b; Suzuki et al., 2013). We could show that medulla neuroblasts in larvae subjected to NR, remain able to transit through the different temporal identities and to generate the corresponding progeny. Although it remains possible that specific neuronal identities, so far unidentified, are skipped in the medulla lineages in response to NR, the fact that all known early and late fates are produced suggests protection of lineage composition to a large extent.

The sparing of medulla lineages may be favored by the timing of their production during development. We have indeed shown that medulla neuroblasts are only converted from the proliferating neuroepithelium once the larva has reached its “critical weight.” In insect, the critical weight is defined as the minimal mass from which the larva can terminate its development in due course without the need of further dietary nutrients. The critical weight triggers the production of a series of ecdysone pulses that progressively commit the larvae to metamorphosis. From this time, larvae confronted to NR rely on their large endogenous nutrient stores to complete their development (Figures 1B,C). We have found that ecdysone production after the critical weight promotes the neuroepithelium-to-neuroblast transition. Therefore, linking the production of neuroblasts in the medulla, and thus the initiation of neurogenesis, to the production of ecdysone, ensures that the larva possesses enough endogenous nutritional reserves to sustain and terminate its development independently of dietary variations. Consistently, neuroepithelial proliferation before the critical weight is highly sensitive to dietary conditions. In principle, this system allows medulla neuroblasts to always benefit from substantial endogenous nutritional reserves and to generate their entire lineage uninterrupted. Yet, the nature of the nutritional reserves involved, as well as the mechanisms promoting their mobilization and their preferential utilization by medulla neuroblasts, remains unclear.

## Modulations of neuronal numbers in the face of nutrient restriction

While sparing strategies ensures that neuroblasts sustain enough growth potential to generate their normal repertoire of progeny independently of nutritional conditions, recent studies have also revealed the surprising plasticity of some regions in their ability to increase or decrease their number of neurons in response to NR. Importantly, in all cases, the neuronal repertoire remains preserved.

### Extrinsically-regulated temporal patterning allows lineage plasticity

**Figure 4:**
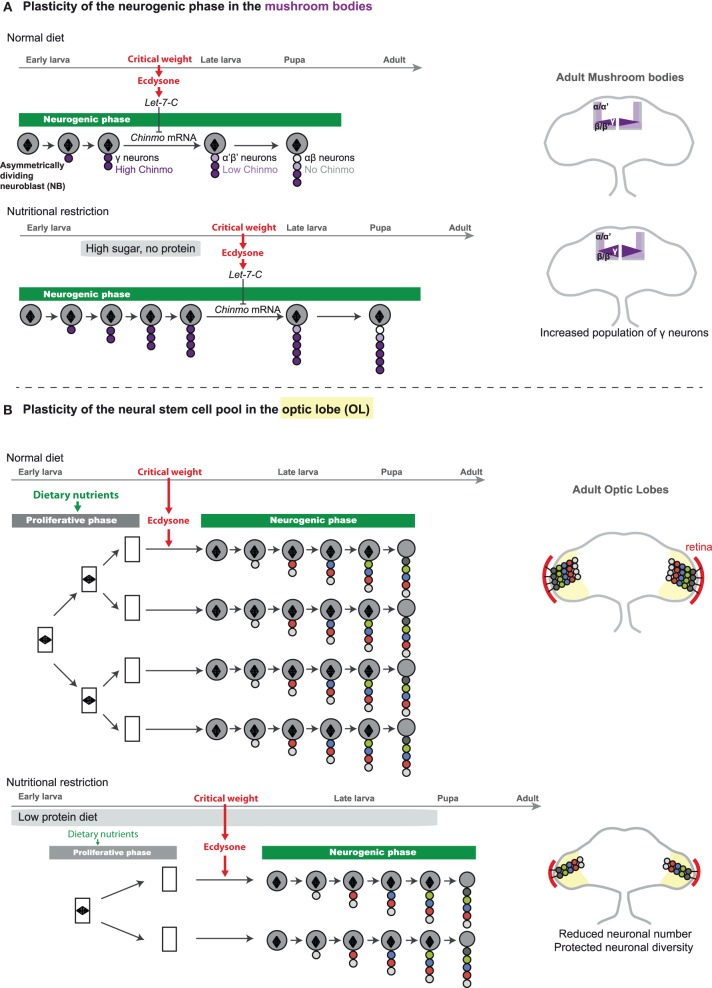
**Adaptative strategies for the modulation of neuronal numbers in the face of nutritional restriction. (A)** In the mushroom bodies (part of the CB), each neuroblast generates a repertoire of neuronal fates, following a stereotypical sequence: γ → α′β′ → αβ. A gradient of Chinmo protein that is expressed in the neuronal progeny dictates the neuronal fate. High levels of Chinmo specify the γ fate, low levels of Chinmo the α′β′, and absence of Chinmo protein is associated with the αβ identity. Mushroom body neuroblasts first generate neurons of the γ fate. Once critical weight is attained, production of ecdysone triggers the expression of micro-RNAs of the *Let-7-Complex* (*Let-7-C*) that target chinmo mRNA, leading to decreasing levels of Chinmo in newly-born neurons. Progressive downregulation of Chinmo over time forces successively born neurons to adopt the α′β′ and finally αβ fate. In a low protein/high sugar diet, the critical weight is delayed, and more neurons of the γ-class are generated (Lin et al., 2013). On the right, schemes represents adult brains with the different classes of mushroom body neurons depicted in various colors. The γ class extends in the high sugar/low protein diet. **(B)** In the optic lobe, neuroepithelium (NE) expansion (proliferative phase) is highly dependent on dietary nutrients. Under poor dietary conditions, a reduced amount of nutritional signals and insulin peptides leads to reduction of NE expansion, and ultimately to a reduced pool of neural stem cells. Proliferating NE cells are represented by rectangles containing a mitotic spindle (diamond shape). Induction of neuroepithelial-to-neuroblast conversion by ecdysone after the critical weight may ensure that asymmetrically-dividing neuroblasts beneficiate from substantial endogenous nutritional stores, allowing lineage integrity under NR. On the right, neurons produced by medulla neuroblasts are represented in the optic lobes of the adult brain under normal or poor dietary conditions. Under NR, neuronal numbers are reduced but their diversity is preserved. The retina is depicted in red and is reduced to similar proportions.

Remarkably, a subset of CB neuroblasts, known as mushroom body neuroblasts, are able to extend part of their lineage when development is delayed upon specific food manipulation (low protein, high sugar diet) (Lin et al., 2013). This lineage plasticity is rooted in the extrinsic mode of temporal specification operating in the mushroom bodies. Indeed, as previously mentioned, the identity of mushroom body neurons presents the particularity not to be specified by an intrinsic timing mechanisms based on the sequential expression of temporal factors in progenitors, but instead depends on external cues. Precisely, mushroom body neuroblasts generate five neuronal sub-classes in a specific temporal order. γ neurons are produced during embryogenesis and early larval stages, α′β′ neurons are produced during late larval stages, and αβ neurons during metamorphosis. Recent work has shown that the transitions between these neuronal sub-classes are regulated by the different pulses of ecdysone produced after the critical weight. Ecdysone promotes in newly born neurons the expression of the *Let-7-Complex* (*Let-7-C*) of microRNAs that downregulate a transcription factor called Chinmo. Chinmo levels in maturing neurons determine their identity (Zhu et al., 2006; Chawla and Sokol, 2012; Kucherenko et al., 2012; Wu et al., 2012). Therefore, temporal transitions in the mushroom body lineages rely on organismal growth and developmental progression. Moreover, mushroom body lineages appear particularly resistant to NR even from early larval stages as, unlike other neuroblasts, they do not undergo quiescence at the end of larval stages and remain active when hatching larvae are protein starved. Consistently, while systemic growth is stalled when early larvae are transferred for several days on a low protein/high sugar diet, mushroom body neuroblasts remain actively dividing and generate more neurons of the γ class, as more time is necessary to reach the critical weight. Thus, the proportions between the different neuronal classes in the mushroom body lineages vary according to nutritional conditions (Figure 4A). Interestingly, parallel investigations on lineages that are controlled by the neuroblast-intrinsic temporal patterning system have revealed that they are not plastic and retain an invariant stoichiometry of neuronal classes under similar nutritional conditions (Kao et al., 2012; Lin et al., 2013).

### Plasticity of the neural stem cell pools

In contrast to the number of neuroblasts in the VNC and CB that are invariant, the number of neuroblasts in the OL strongly depends on dietary conditions. Indeed, it appears that the proliferative phase of neuroepithelium development, that occurs during the first two-third of larval stages (before the critical weight), is highly sensitive to the Insulin and TOR pathways whose activities depend on dietary conditions. Consequently, larvae that have been continuously raised in a suboptimal diet (consisting in the usual lab medium diluted 10 times), exhibit a delayed development and a reduced number of neuroblasts at the end of larval stages due to a lower rate of neuroepithelial proliferation prior to the ecdysone pulse (Figure 4B). This leads to adult flies that possess fewer lineages and therefore fewer neurons in their visual system. Yet this adaptive strategy preserves neuronal diversity as neuroblast lineages do not vary upon NR. Consequently the number of medulla units is reduced but their functionality is preserved. As the number of photoreceptors projecting to the medulla is also reduced due to a lower number of units in the fly retina, this system could account for preservation of retinotopic mapping in smaller flies with smaller eyes (Figure 4B).

## Principles of sparing and plasticity during drosophila CNS development

From this set of recent studies, a few key principles have emerged that govern brain sparing and plasticity in *Drosophila*:

- Neuroblast asymmetric divisions are globally spared against NR. Protection is achieved through the existence of alternative growth pathways in progenitors, such as ALK, that do not depend on InR and TOR (Figure 3). Moreover, the confinement of large periods of neurogenesis during developmental windows with high endogenous nutritional reserves (embryogenesis or stages after the critical weight) provides the necessary fuel for sustaining neuroblast growth and divisions when dietary nutrients are scarce (Figure 1B). Although there are so far no examples of precocious neuroblast exhaustion and truncated lineages, it remains formally possible that some lineages are more sensitive and skip-specific neuronal fates when nutrient are lacking. Further studies and the discovery of novel markers will tell.
- Lineages, such as those in the mushroom bodies, in which temporal identity transitions are controlled by extrinsic cues linked to organismal growth, are highly plastic and the relative proportions of the different neuronal subclasses vary according to nutritional conditions (Figure 4A). In contrast, the composition of lineages in which temporal identity transitions are governed by progenitor-intrinsic transcriptional programs appears fixed.
- Plasticity in the number of neurons can be achieved without affecting neuronal diversity through the regulation of uniform pools of NSCs. In the OL, the proliferative phase of the neuroepithelium happens before the critical weight, and is highly sensitive to Insulin and amino acids. Consequently, a poor diet leads to a reduced pool of neuroepithelial cells. Ultimately, fewer neuroepithelial cells are converted in fewer neuroblasts leading to a reduction in the final number of neuronal lineages (Figures 1C, 4B).

Following these principles, different regions of the brain exhibit different adaptive properties consistent with their neurological function.

Many CB and VNC lineages are unique and utilize all protection mechanisms. This ensures that irreplaceable lineages are produced with minimal interference from nutritional conditions. Mushroom body lineages are also spared, but their mode of temporal specification allows plasticity, and the matching of their neuronal composition with nutritional conditions. Given the role of the mushroom bodies in memory and learning capacity in response to olfactory cues, the increase of γ neurons under low protein/high sugar diets suggests an adaptive mechanism that expands the developing olfactory system as amino-acid resources become scarce. Interestingly such diets are characteristic of modern societies, and future studies should address their impacts on the neuronal composition of mammalian brains.

Finally, in the OL, the medulla is composed of multiple lineages of similar composition. In this situation, reduction of the NSC pool upon NR offers the possibility of limiting the energetic cost of the late, and protected, neurogenic phase, and of privileging the quality of lineages being generated rather than their number. Moreover, given that the OL represents a large proportion of the adult brain, the subsequent reduction of the visual system represents an adaptation to continued adverse nutritional conditions by substantially decreasing energetic consumption during adulthood.

Together, these studies have revealed a sparing hierarchy among the different regions of the brain allowing a balanced coping strategy with the goal of preserving functionality and adapting the adult nervous system to suboptimal nutritional conditions.

## Conclusion

Thanks to a detailed knowledge of developmental mechanisms, the ability to conveniently manipulate food intake, and the vast repertoire of genetic tools, it is now possible to uncover the basic principles that govern sparing and plasticity in the developing *Drosophila* brain under conditions of NR. This work paves the way for an integrated understanding of the molecular, cellular, and organismal mechanisms controlling brain growth. In the future, it will be important to investigate whether similar growth-boosting pathways and region-specific adaptive strategies are conserved in other organisms and may ultimately apply to the development of the embryonic and fetal mammalian brain. *Drosophila* may also become a successful model to investigate the long-term consequences of developmental NR on behavior and brain ageing.

Uncovering the mechanistic basis of brain protection, as well as identifying its weaknesses, could help to define therapeutic strategies reducing the negative effects of prenatal undernourishment and improving brain recovery after birth. In this field of research, as in many others, *Drosophila* will doubtless continue to make its discrete but pioneering contribution.

### Conflict of interest statement

The authors declare that the research was conducted in the absence of any commercial or financial relationships that could be construed as a potential conflict of interest.
